# Optimizing (O) rifapentine-based (RI) regimen and shortening (EN) the treatment of drug-susceptible tuberculosis (T) (ORIENT) using an adaptive seamless design: study protocol of a multicenter randomized controlled trial

**DOI:** 10.1186/s12879-023-08264-2

**Published:** 2023-05-08

**Authors:** Zhen Feng, Yan Miao, Ying Peng, Feng Sun, Yilin Zhang, Rong Li, Shijia Ge, Xinchang Chen, Lingyun Song, Yang Li, Xiaomeng Wang, Wenhong Zhang

**Affiliations:** 1 Department of Infectious Diseases, Shanghai Key Laboratory of Infectious Diseases and Biosafety Emergency Response, National Medical Center for Infectious Diseases, Huashan Hospital, Shanghai Medical College, Fudan University, Shanghai, China; 2 Shanghai Huashen Institute of Microbes and Infections, Shanghai, China; 3 Department of Tuberculosis Control and Prevention, Zhejiang Provincial Center for Disease Control and Prevention, Zhejiang Province, Hangzhou, People’s Republic of China

**Keywords:** Tuberculosis, Short-course regimen, Drug-susceptible tuberculosis, Randomized controlled trial, Non-inferiority

## Abstract

**Background:**

Standard treatment for drug-susceptible tuberculosis (DS-TB) includes a multidrug regimen requiring at least 6 months of treatment, and this lengthy treatment easily leads to poor adherence. There is an urgent need to simplify and shorten treatment regimens to reduce interruption and adverse event rates, improve compliance, and reduce costs.

**Methods:**

ORIENT is a multicenter, randomized controlled, open-label, phase II/III, non-inferiority trial involving DS-TB patients to evaluate the safety and efficacy of short-term regimens compared with the standardized six-month treatment regimen. In stage 1, corresponding to a phase II trial, a total of 400 patients are randomly divided into four arms, stratified by site and the presence of lung cavitation. Investigational arms include 3 short-term regimens with rifapentine 10 mg/kg, 15 mg/kg, and 20 mg/kg, while the control arm uses the standardized six-month treatment regimen. A combination of rifapentine, isoniazid, pyrazinamide, and moxifloxacin is administered for 17 or 26 weeks in rifapentine arms, while a 26-week regimen containing rifampicin, isoniazid, pyrazinamide, and ethambutol is applied in the control arm. After the safety and preliminary effectiveness analysis of patients in stage 1, the control arm and the investigational arm meeting the conditions will enter into stage 2, which is equivalent to a phase III trial and will be expanded to recruit DS-TB patients. If all investigational arms do not meet the safety conditions, stage 2 will be canceled. In stage 1, the primary safety endpoint is permanent regimen discontinuation at 8 weeks after the first dose. The primary efficacy endpoint is the proportion of favorable outcomes at 78 weeks after the first dose for both two stages.

**Discussion:**

This trial will contribute to the optimal dose of rifapentine in the Chinese population and suggest the feasibility of the short-course treatment regimen containing high-dose rifapentine and moxifloxacin for DS-TB.

**Trial registration:**

The trial has been registered on ClinicalTrials.gov on 28 May 2022 with the identifier NCT05401071.

**Supplementary Information:**

The online version contains supplementary material available at 10.1186/s12879-023-08264-2.

## Background

Tuberculosis (TB) is one of the most important global healthcare challenges, and is one of the leading causes of death from infectious pathogens, with 10.6 million people contracting TB and 1.6 million dying from it in 2021 [1]. Currently, the course of chemotherapy for the initial treatment of drug-susceptible tuberculosis (DS-TB) is 6 months, which is much longer than that of other respiratory diseases [2]. Minimal non-adherence to TB treatment was associated with an increased risk for unfavorable outcomes [3]. The development of new and safe therapeutic strategies with potent bactericidal activity is the main approach to short treatment duration, improve compliance and ensure the success rate, thus reducing the burden of both doctors and patients [4].

On the strength of their powerful bactericidal and sterilizing activities, rifamycins are the cornerstone of modern chemotherapy for active TB with a 6-month course and play a vital role in preventing recurrence after cessation of treatment [5]. Rifampicin and rifapentine are the two forms of rifamycins. Rifampicin is the most commonly used rifamycin in the treatment of TB. The association between rifampicin exposure and antimicrobial activity was confirmed in previous phase I and II clinical trials, as assessed by early bactericidal activity and sputum culture conversion time, and the plateau of rifampicin activity was not achieved even at doses as high as 35 mg/kg/d [6–8]. As the cyclopentyl derivative of rifampicin, rifapentine has a longer half-life and a lower minimum inhibitory concentration than rifampicin [9, 10]. Therefore, the potential of rifapentine in intensifying treatment was explored [11–13]. The use of higher and more frequent doses of rifapentine was shown to achieve higher cure rates with a satisfactory safety profile, even at doses up to 20 mg/kg/day [14–18].

Apart from high-dose rifapentine, using fluoroquinolones in constructing a new regimen is thought to probably contribute to shortening the treatment duration of DS-TB [19]. However, it is worth noting that compared with the standard treatment, 4-month fluoroquinolone-contained regimens were shown inferior in three large trials in 2014 [20–22], which implied fluoroquinolones alone were not sufficient to shorten the course of treatment [23]. An animal study conducted in 2007 showed that combined treatment with rifapentine and moxifloxacin showed high early bactericidal activity in a mouse model of TB and provided a high cure rate after 3 months of treatment [24].

Since then, the combination of fluoroquinolones and higher doses of rifapentine has received considerable attention. The results of a multicenter randomized controlled trial S31/A5349, published in the New England Journal of Medicine in April 2021, showed that replacing rifampicin with a high dose of rifapentine (1200 mg daily) and substituting moxifloxacin for ethambutol could shorten the treatment period of DS-TB to 4 months without increasing the occurrence of adverse events (AEs). This strategy proved to be non-inferior to the standard regimen and was recommended by the World Health Organization (WHO) in May 2022 [2, 25].

Enthusiasm over the promising four-month regimen based on high-dose rifapentine and moxifloxacin has been curtailed following clinicians’ concerns about potential hepatotoxicity as the dose of rifapentine in the recommended regimen is much higher than that approved by the US Food and Drug Administration (FDA) and the China National Medical Product Administration (NMPA). Therefore, it is imperative to continuously evaluate the safety profile and efficacy of the high-dose rifapentine-based regimen among expanded populations and explore the possibility and practicability of optimizing the dose of rifapentine without compromising efficacy.

To delight this uncertain, we propose a phase II/III, multicenter, open-label, noninferiority, randomized clinical trial, entitled “Optimizing RIfapentine-based regimen and shortENing the treatment of Tuberculosis research (ORIENT)”, aiming to evaluate whether the 4-month regimen containing high-dose rifapentine and moxifloxacin is as effective and safe as the 6-month standard regimen for DS-TB in Chinese people.

The effectiveness, safety, and tolerability of a new four-drug therapy for 17 weeks will be explored in comparison with the standard 6-month treatment for DS-TB. At the same time, considering some characteristics that may have an impact on drug efficacy, the pharmacokinetic/pharmacodynamic (PK/PD) analysis of rifapentine and its desacetyl metabolite and moxifloxacin will be performed. And the protocol of this clinical trial is presented here.

## Method

### Study objectives

ORIENT is a phase II/III, multicenter, open-label, randomized controlled clinical trial of non-inferiority design, recruiting DS-TB patients susceptible to rifampicin, isoniazid, pyrazinamide, ethambutol, and fluoroquinolones. The purpose of this study is to a) Evaluate the safety, tolerability, and PK/PD of a high-dose rifapentine regimen; b) Evaluate whether the regimen containing high-dose rifapentine and moxifloxacin may reduce the duration of DS-TB treatment to 17 weeks; c) define the optimal dose of rifapentine in the Chinese population.

### Study design

**Fig. 1:**
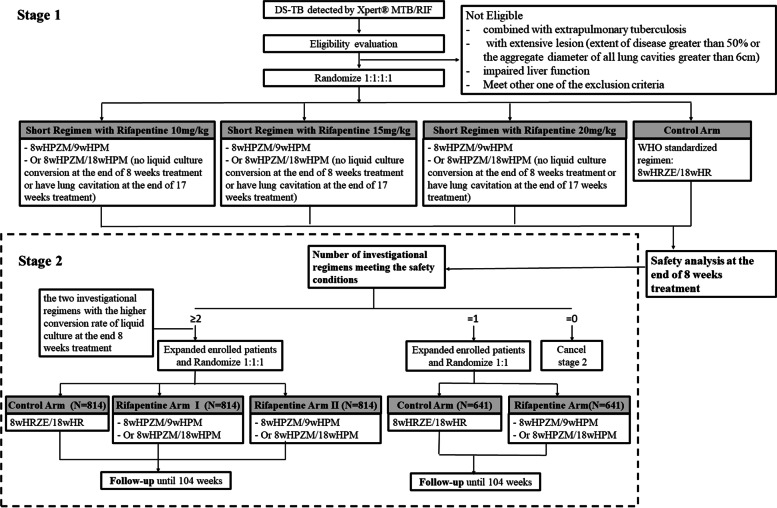
ORIENT study schematic. The trial plans to recruit DS-TB patients susceptible to rifampicin, isoniazid, pyrazinamide, ethambutol, and fluoroquinolones. Participants will be randomly divided into different arms as shown in the figure. Abbreviations in this figure: DS-TB: drug-susceptible tuberculosis. 8wHRZE/18wHR: rifampicin (R), isoniazid (H), pyrazinamide (Z), and ethambutol (E) for 8-week intensive phase and rifampicin and isoniazid for 18-week continuous phase. 8wHPZM/9wHPM: rifapentine (P), isoniazid(H), pyrazinamide (Z), and moxifloxacin (M) for 8-week intensive phase and rifapentine, isoniazid and moxifloxacin for 9-week continuous phase. 8wHPZM/18wHPM: rifapentine (P), isoniazid (H), pyrazinamide (Z), and moxifloxacin (M) for 8-week intensive phase and rifapentine, isoniazid and moxifloxacin for 18-week continuous phase

The study is composed of two stages in a seamless progression, which means only after the decision has been taken following the analysis of stage 1 primary endpoint, the participant recruitment of stage 2 will start. Figure 1 shows the flowchart of ORIENT trial. The WHO standardized regimen for DS-TB is used as a control arm for two stages.

Stage 1 corresponds to the phase II trial, aiming to evaluate the safety of investigational regimens in patients with DS-TB. Recruited participants are randomly assigned to the Short Regimen with Rifapentine 10 mg/kg, Short Regimen with Rifapentine 15 mg/kg, and Short Regimen with Rifapentine 20 mg/kg as investigational arms and the WHO Standardized Regimen as a control arm.

If at least two investigational arms meet the safety conditions in the stage 1 analysis, the two investigational arms with the higher conversion rate of liquid culture in 8 weeks and the control arm will enter stage 2. The subsequently enrolled patients will be expanded and be allocated to the 3 arms at a random ratio of 1:1:1. If only one arm meets the safety conditions in stage 1, the participants with DS-TB will be expanded and be 1:1 randomly assigned to this certain investigational arm and the control arm. If all investigational arms in stage 1 fail to meet the safety conditions, stage 2 will be canceled.

### Study regimens and comparator

The rifapentine regimens are composed of two periods of 17 to 26 weeks. The first phase is an 8-week intensive phase, including rifapentine (P), isoniazid (H), pyrazinamide (Z), and moxifloxacin (M) for daily use. This is followed by a 9-week continuation phase with the following agents every day: rifapentine, isoniazid, and moxifloxacin (8wHPZM/9wHPM) (The continuation phase should be extended to maximum of 18 weeks for patients who have lung cavitation at the end of 17 weeks treatment on chest radiography or positive sputum culture in liquid medium at the end of 8 weeks treatment (8wHPZM/18wHPM)). The control regimen is the WHO standardized regimen with two phases of treatment for 26 weeks. The first is an intensive phase using rifampicin, isoniazid, pyrazinamide, and ethambutol (E) every day for 8 weeks. This is followed by a continuation phase of 18 weeks with the following agents for daily use: rifampicin and isoniazid (8wHRZE/18wHR). Table 1 shows the doses and usage of each drug in the WHO standardized regimen and the short regimen with rifapentine.

**Table 1:**
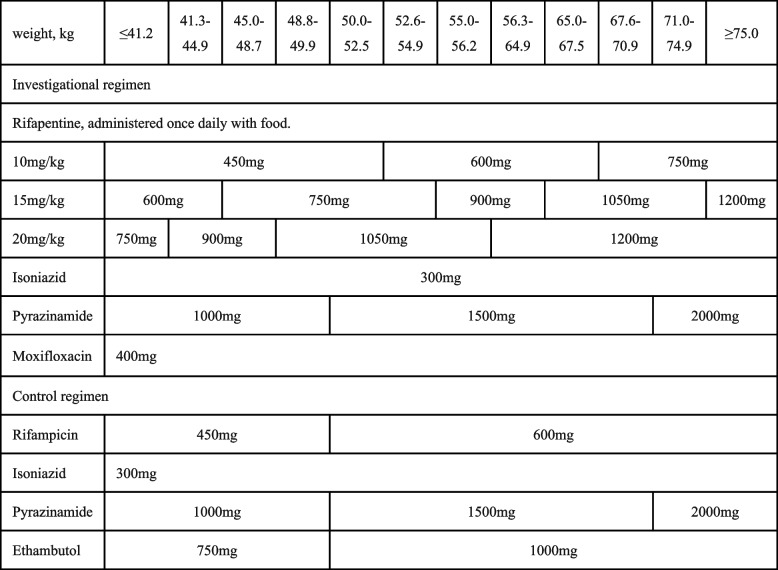
Doses and usage of study drugs by body weight

All drugs will be administered orally

Rifampicin, isoniazid and pyrazinamide are given once a day before breakfast

Moxifloxacin and rifapentine should be administered together once daily with food

Ethambutol should be taken by body weight 750 to 1000 mg/d, divided into 1 to 3 times, before or after meals

### Enrollment termination criteria and safety conditions in stage 1 analysis

The primary aim of stage 1 is to choose drug regimens for the assessment of stage 2, based on the 8-week efficacy and safety endpoint evaluation. Any study arm will be terminated early when any of the following conditions are present: 1) The number of patients with serious adverse events(SAEs) related to the study drug is more than 2, and the proportion exceeds 20% of the number of enrolled people in this arm; 2) More than 1 case of liver failure or death related to the study drug and the proportion exceeds 10% of the enrolled population in this arm; 3) Other situations require discontinuation of arm decided by the Safety Reviewer Committee (SRC).

Stage 2, equivalent to a phase III trial, will expand subsequent enrollment, if the safety evaluation in stage 1 meets all the following three conditions:1) The arm has not stopped in advance due to safety; 2) The trial has been assessed as tolerable (defined as the upper bound of the 90% one-sided confidence interval of the percentage of participants permanently discontinuing treatment < 30%); 3) There is no other situation that warrants discontinuation of the study arm after the decision by the SRC.

### Site selection

This trail is under the guidance of the China National Tuberculosis Defense Association, sponsored by the National Medical Center for Infectious Diseases (NMCID is a national medical center established based on Huashan Hospital, Fudan University, aiming to create a highland of medical treatment technology and improve the overall and regional medical service capacity of the country). The recruiting-cooperative units are 12 hospitals in four provinces in China, namely Huashan Hospital, Hangzhou Red Cross Hospital, Wenzhou Central Hospital, Hunan Chest Hospital, First People's Hospital of Hangzhou Xiaoshan District, First People's Hospital of Linping District, Guiyang Public Health Clinical Center, The Third People’s Hospital of Bijie City, People’s Hospital of Anshun City Guizhou Province, Guizhou Aerospace Hospital, Liupanshui Third People's Hospital, Affiliated Hospital of Zunyi Medical University. The special audit team will carry out quality control for the cooperative unit every month.

### Patient eligibility criteria

In addition to volunteering to participate in this clinical trial, the main eligibility criterion is pulmonary TB caused by *Mycobacterium tuberculosis* susceptible to rifampicin detected by molecular or phenotypic susceptibility testing. Participants must have a respiratory specimen, that is either positive for acid-fast bacilli on smear microscopy or positive for *Mycobacterium tuberculosis* by Xpert® MTB/RIF (Cepheid, Sunnyvale, CA, USA). Besides, patients aged between 18 to 60 years and weighed between 40 to 80 kg are eligible for the trial. An individual meeting any of the following exclusion criteria will be excluded from screening: resistance to isoniazid, pyrazinamide, ethambutol, or fluoroquinolones, and patients with extrapulmonary TB or with an extensive lesion (extent of disease greater than 50% or the aggregate diameter of all lung cavities greater than 6 cm). In addition, considering the safety of the regimen, patients will be excluded if they suffer from impaired liver function, their estimated glomerular filtration rate is smaller than 90 mL/min/1.73m^2^, or live with human immunodeficiency virus (HIV). Patients will also be excluded if they are taking drugs that affected the efficacy or contraindications with this study drugs. Moreover, pregnant or breastfeeding women are excluded. Detailed inclusion and exclusion criteria are shown in Table 2.

**Table 2:** ORIENT inclusion and exclusion criteria

	**Detailed Description**
Inclusion criteria	1) Willing and able to participate in this trial, and signed informed consent (sign by the legal representative if patient has no civil capacity)
	2) Aged of 18–60 years old
	3) Weighed of 40–80 kg 4) Respiratory specimen positive for acid-fast bacilli on smear microscopy or positive for *M. tuberculosis* by Xpert® MTB/RIF (Cepheid, Sunnyvale, CA, USA)
	5) Sensitive to rifampicin by molecular or phenotypic susceptibility testing
	6) Has not received any anti-TB treatment in the past 6 months
	7) If female patient not pregnant, willing to apply effective contraception during treatment
Exclusion criteria	1) Combined extrapulmonary tuberculosis
	2) Extensive lesion (extent of disease greater than 50% or the aggregate diameter of all cavities greater than 6 cm) 3) *M. tuberculosis* isolate is already known to be resistant to at least one of the following: isoniazid, pyrazinamide, ethambutol, or fluoroquinolones
	4) Patients with impaired liver function (Alanine aminotransferase or alkaline phosphatase or total bilirubin is higher than 1.5 times the upper limit of normal; hepatic encephalopathy, ascites)
	5) White blood cell is less than normal or hemoglobin is less than 90 g/L or platelet is less than 100*10^9/L
	6) Estimated glomerular filtration rate is less than 90 mL/min/1.73m^2^
	7) HIV antibody positive and AIDS patients
	8) Allergy or intolerance to any study drugs
	9) Unable to take oral medications
	10) If the following drugs are currently or are planned to be used within 6 months of enrollment: quinidine, procaine, amiodarone, sotalol, propylamine, zilasidone or tephenadine
	11) Known history of optic neuritis, alcohol abuse, prolonged QT syndrome or QTcF over 450 ms at baseline, epilepsy, mental illness, diabetes with fundus disease, gout, porphyria, myasthenia gravis
	12) Pregnant or breastfeeding

### Recruitment process

DS-TB patients identified by Xpert® MTB/RIF are being screened to determine whether they meet other eligibility criteria for inclusion. The screening information in this trial includes medical history, signed informed consent, results of physical examination, clinical evaluation, sputum smear, Xpert® MTB/RIF, liver function, renal function, blood routine, blood glucose, blood potassium, hepatitis B and C, HIV testing, pregnancy testing, electrocardiogram, chest radiography, and ophthalmologic examination, including assessment of visual acuity and color vision. After screening procedures, these eligible patients without evidence of resistance to isoniazid, pyrazinamide, ethambutol, and fluoroquinolones will be recruited into this trial.

### Treatment allocation

Online registers will be applied to the patients satisfying the eligibility criteria. To balance the bias of each center from different regions, the randomization is conducted through the online central randomization system stratified by the study sites (sponsored by REDCap) and the presence of lung cavitation. In stage 1, participants will be 1:1:1:1 randomly assigned to the study regimens. In stage 2, if 3 regimens are enrolled, participants will be 1:1:1 randomly assigned into the 3 arms; If 2 arms are enrolled, participants will be assigned with a random ratio of 1:1.

### Duration of follow-up

After screening and baseline assessment, follow-up visits will be conducted every 3 days to 2 weeks in the 8-week intensive phase, every 4 to 5 weeks until the end of treatment, every 12 to 14 weeks at the period of post-treatment until 78 weeks after treatment initiation, and the last follow-up visit is 104 weeks after treatment initiation. All patients undergo intensive monitoring of liver function and blood routine, especially for the first two weeks after the first dose. During the follow-up, physical examination, clinical assessment, weight measurement, sputum smear, sputum culture, blood routine, liver function, renal function, electrocardiogram, ophthalmologic examination, and other adjuvant examinations will be done according to schedule (the Additional file). Study enrollment began in November 2022.

### Sample size assumptions

According to previous studies, the percentage of participants with regimen discontinuation at 8 weeks after the first dose is estimated to be 20%. Using the PASS 15 system (NCSS, Version: 15.0.5), we assumed α = 0.1 and β = 0.2 and calculated that a sample size of 96 cases in each group was required for safety assessment in stage 1. Considering a 4% lost follow-up rate, the number of cases per arm to show safety was calculated as 100 (400 in total).

This sample size of stage 2 was also calculated using the PASS 15 system. Assuming α = 0.05, β = 0.2, according to previous studies, the success rate of the control group was 85.4%, and the success rate of the test group was 84.5%, σ = 6.6%. If stage 2 included three arms for research, according to Bonferroni correction α_1_ = α/2 = 0.025, according to PASS 15 non-inferiority test system, 618 samples were required for each arm. In consideration of 12% of patients not satisfying the criteria for microbiological eligibility and 12% of patients not being assessed, 814 patients were required for each arm, so the total sample size of the two arms was considered to be 2442. If stage 2 was divided into two arms for the study, the significance level was set as α = 0.05 with no correction required. According to the PASS15 non-inferiority test system, 487 samples per arm were required. Considering that 12% of patients did not meet the microbiological eligibility criteria and 12% of patients could not be evaluated, thus the total number was considered to be 1282 with 641 samples needed per group.

### Data collection and quality management

Dedicated websites and data collection forms will be applied by collaborating hospitals or centers for research management to record data. Unique usernames and passwords will control all data access on websites, so staff only have access to the functionality and data that are appropriate for their role in the study restrictedly. The responsibility of the Central Coordinating Office (CCO) staff will be the provision of the relevant website and case report forms. The eligibility of patients and follow-up data completeness will be also checked by CCO regularly. The database will be locked after the completion of the whole follow-up schedule and data review.

### Adverse event management

SRC is established in this trial to review and discuss the existing safety and efficacy data to determine whether the arm is suitable to continue to the next stage and provide professional advice on clinical management strategy. SRC is composed of a powerful clinical team of TB specialists, pulmonologists, infectious disease experts, nephrologists, psychiatrists, cardiologists, hematologists, and rheumatologists. SRC meetings are divided into regular and urgent meetings. Regular SRC meetings will be held for routine safety data review every 2 months. Any SAE must be reported by investigators to the SRC within 24 h, meanwhile, the urgent SRC meeting will be triggered to decide the execution of the related cases and treatment arms, including adjustment of anti-TB regimens, discontinuation of treatment arms, etc. AEs are defined and reported by the site investigator according to the Common Terminology Criteria for Adverse Events 5.0 (CTCAE) [26]. An SAE is classified if it led to death, life threatening, prolonged hospitalization, permanent or serious disability, malformation or congenital defect of the offspring, or required hospital admission for management. All AEs and the adjustment or withdrawal of drugs in this trial will be recorded and checked by CCO.

### Assessment and analysis of outcome

The primary safety outcome of stage 1 is the proportion of participants with regimen discontinuation for safety reasons at the end of the first 8 weeks of treatment (regimen discontinuation is defined as greater than or equal to one drug permanent withdrawal from the assigned regimen). The safety outcomes measured in both stages include the AEs and SAEs assessment, the proportion of participants with grade 3 or higher AEs during the period of treatment.

The primary efficacy endpoint is the proportion of favorable outcomes at 78 weeks after the first dose for both stages. Besides, the secondary outcomes of stage 1 include liquid culture conversion rate at the 8 weeks of treatment, the proportion of participants with favorable outcomes at 104 weeks after the first dose, and incidence of acquired resistance during treatment and follow-up. The secondary outcomes of stage 2 include the percentage of participants with favorable outcomes at 104 weeks after the first dose, recurrence rate after treatment completion, proportion of sputum solid and liquid culture conversion at 8 weeks and at the end of the treatment, median time of sputum solid and liquid culture conversion, and rate of acquired resistance during treatment and follow-up.

For each participant, treatment outcomes includes favorable outcome, unfavorable outcome and not assessable outcome, as described in Table 3.

**Table 3:** Definition of the favorable and unfavorable outcomes in ORIENT study

Outcome	Description
Favorable outcome	The outcome will be classified as favorable if not classified as unfavorable or not assessable and meets any one of the following criteria
	1) The sputum culture is negative for *M. tuberculosis* at the end of follow-up period 2) The sputum can be contaminated or no longer be produced at the end of follow-up period, without evidence of active pulmonary tuberculosis after a stable sputum culture conversion^a^ has been achieved
Unfavorable outcome	The outcome will be classified as unfavorable if:
	1) Sputum culture is positive for *M. tuberculosis* for two consecutive times after treatment; And the second strain is confirmed indistinguishable from the original isolate by the whole genome sequencing. If the whole genome sequencing result is not available, it defaults to the same strain
	2) The sputum culture at the last visit of follow-up is positive for *M. tuberculosis*
	3) Participants die during treatment (excluding force majeure factors such as homicide, traffic accidents or natural disasters); death associated with tuberculosis during follow-up (after completion of study treatment)
	4) Withdrawal from the trial or loss to follow-up during treatment (except pregnancy, accident, etc.)
	5) Participants stop the initial treatment, and restart different tuberculosis treatments
	6) Restart tuberculosis treatment after the end of expected treatment
	7) The treatment course exceeds the expected treatment length by more than 2 weeks regardless of any reason
	8) The treatment regimen is changed (including dosage or frequency of administration) except for pregnancy, exogenous reinfection, and rechallenge
Not assessable	The outcome will be classified as not assessable if not classified as unfavorable and meets any one of the following criteria: 1) Loss to follow-up (after completion of study treatment) with the last sputum culture being negative; 2) Sputum cannot be produced or is contaminated at the end of follow-up period, but stable culture conversion has not been achieved before and the last sputum culture is negative; 3) Alteration of treatment due to pregnancy; 4) Death unrelated to tuberculosis during follow-up (after completion of study treatment); 5) Death due to force majeure such as homicide, car accident or natural disaster during treatment; 6) Extension or change of tuberculosis treatment after the whole genome sequencing confirms exogenous reinfection

^a^Stable culture conversion is defined that the subject shows negative culture of *M. tuberculosis* in two consecutive samples (at least 2 weeks apart) for the first time, and subsequently no positive culture of *M. tuberculosis* or only one single positive (at least two consecutive bacterial cultures after the definition of the positive strain are negative, and no evidence of exogenous reinfection is found after sequencing of the positive strain) is shown

All subjects who have taken at least one dose of the treatment will be included in the safety analysis. The analysis for efficacy in this study will be conducted with modified intention-to-treat (mITT), assessable, and per-protocol populations with primary consideration for mITT results. For the primary outcome measurement in stage 2, the upper bound of the 95% confidence interval for the difference in favorable outcome between the control arm and the rifapentine arms must be less than 6.6% (the margin of non-inferiority) in the mITT populations for the rifapentine regimen to be declared non-inferior to the WHO standardized regimen.

### Pharmacokinetic sampling

Sampling for pharmacokinetic analysis of anti-TB drugs is performed for all participants with rifapentine regimens in stage 1. Pharmacokinetic sampling is divided into intensive and sparse sampling, and 20 to 30 people in each rifapentine group perform intensive sampling and all other participants undergo sparse sampling.

An intensive sampling scheme includes two samplings. The first intensive sampling is at the first dose after randomization, and 4 ml venous blood is obtained at 0 h before administration and 0.5 h, 1 h, 2 h, 4 h, 6 h, 8 h, 12 h, 24 h, 48 h, 72 h, and 96 h after administration, respectively. The second intensive sampling will be permitted at any time between the 11th study drug dose and the week 8 visit, and blood samples, 4 ml each, is collected over a 96-h period at the same time as the first intensive sampling. Subjects do not take any anti-TB drugs during the intensive sampling period. For patients participating in intensive sampling, the first administration is not included in the course of treatment, meaning that the treatment period begins with completing the first sampling (the fourth day of the first sampling).

Patients participating in sparse sampling are sampled during routine follow-ups between week 2 and week 8 visits. The volume of venous blood collected at each follow-up for sparse sampling is approximately 4 ml, and the collection time will be recorded. Blood specimens will be shipped to the designated laboratory, in which concentrations of moxifloxacin, rifapentine, and metabolites of rifapentine are measured.

### Confidentiality

The personal information of participants is restricted to information required for the assessment of trial outcomes with laws on privacy protection and guaranteeing confidentiality. Cooperative hospitals need to have an exclusive office to lock paper documents recording participants’ data. We also conduct password-protected files to store digital documents on the website. Access to these research files is restricted to authorized personnel only.

## Discussion

Treatment shortening is a practical and implementable option in TB control [27, 28]. Over the past few decades, more than 90 countries have been using the standard six-month treatment regimen, despite the emergence of many new anti-TB drugs [5, 29]. Despite the enormous challenges involved in the optimization of DS-TB treatment, there is no doubt that the future success of TB eradication will depend on rapid and accurate drug susceptibility testing, stratified approaches to TB therapeutics, and short-course treatment regimens [19, 30]. In recent years, the number of clinical trials in the DS-TB field has increased as the concept of short-course treatment has already spread, and the 4-month regimen has revolutionized DS-TB treatment guidelines [19, 25, 31–33]. In May 2022, the latest WHO guidelines recommended the use of a 4-month regimen consisting of high-dose rifapentine, isoniazid, pyrazinamide, and moxifloxacin for the treatment of DS-TB in patients 12 years of age and older [2]. Then, considering the reality of each country, how to deal with the treatment and public health strategy changes brought about by the 4-month regimen has become the tasks that clinicians and public health experts need to face together. And to solve this problem, we started this multicenter clinical trial in China.

ORIENT is a multi-arm, multi-stage, multicenter, phase II/III, non-inferiority clinical trial aiming to identify safe and efficacious regimens to treat DS-TB and determine optimal rifapentine dosing in Chinese people. The adaptive trial design is chosen to evaluate a range of candidate regimens containing different doses of rifapentine and to ensure that the most promising regimen(s) into stage 2 with a seamless transition. Besides, patients enrolled in stage 1 of the trial will also be followed through to week 104 after the first dose and the findings will contribute to the stage 2 sample size calculation. This trial design is intended to deliver a shorter and more effective treatment regimen to high-burden countries as soon as possible.

The safety concern of the short regimen WHO recommended is the dose of rifapentine, 1200 mg for daily use, which is far more than the dose prescribed by the FDA and NMPA. This is easy to limit the application of a high-dose rifapentine regimen. Further clinical trials are warranted to determine the safety of rifapentine at a dose of 1200 mg daily. This will be the key issue to be addressed when scaling up the 4-month treatment regimen in the future. With the consideration of the potential risk of this study, SRC is established to assist in the management of AEs and SAEs through regular and urgent meetings.

Notably, pharmacokinetic data will be available in the trial, and population pharmacokinetics will be integrated into the phase II clinical trial to address differences in response across geographic regions and populations. There is few pharmacokinetic data on high-dose rifapentine in Chinese TB patients. Our research is expected to fill this gap. In addition, one phase II clinical trial conducted by Savic and colleagues investigated the relationship between rifapentine exposure and treatment response in TB patients given high doses of rifapentine daily [18]. However, only 34 participants received a rifapentine dose of 1200 mg in Savic's trial, so more pharmacokinetic studies with larger sample sizes receiving different higher doses of rifapentine warrant further investigation.

To further optimize the design and management of short-course regimens for DS-TB patients, this trial rises the drug susceptibility testing (DST) for fluoroquinolone to the same level as isoniazid and rifampicin**,** when moxifloxacin is upgraded to a first-line drug. Fluoroquinolones are widely used in common infections, such as respiratory tracts, wounds, urinary and gastrointestinal tracts, and other infections. In countries with a high TB burden, large numbers of undiagnosed TB cases may take fluoroquinolones, which can lead to the emergence of resistance in *Mycobacterium tuberculosis* [34, 35]. The fifth national TB epidemiological survey of China in 2010 suggested that moxifloxacin-resistant isolates detected accounted for 7.4% of new TB patients [36, 37]. The shorter the course of treatment, the greater the need for individual drug effectiveness. Therefore, to reduce relapse rates and optimize patients’ treatment process, it is vital to emphasize the importance of DST for fluoroquinolone to ensure the drug’s effect when fluoroquinolone is applied in the shortened treatment of DS-TB.

The pursuit of maximum cure rates for TB is a vital public health priority and maybe even more important than reducing the length of treatment, as recent modeling work has shown that improved treatment will have the greatest effect in reducing mortality and the burden of disease worldwide [3, 38]. In actual clinical management, the key to a better treatment outcome lies in the time of the drug withdrawal decided by the clinician. However, clinicians sometimes have difficulties in grasping the course of treatment flexibly. In our study, the total duration of treatment will be individualized according to the culture conversion in liquid medium at the end of 8 weeks and the lung cavitation in chest radiography at the 17 weeks of treatment, which will reflect treatment response. Therefore, ORIENT can provide evidence for a clinician to decide the course of treatment and not have to stick to it for a certain duration. And based on a comprehensive evaluation of the treatment effects on patients, it is possible to find a balance between individual clinical treatment and the short-course chemotherapy strategy in the future.

Limitations include restricted generalizability to certain groups of people such as people under 18, old people over 60, pregnant women, and people living with HIV, who are excluded from the trial mainly for safety considerations. Besides, we did not perform a phase II clinical trial to explore the safety of high-dose rifapentine in Chinese healthy people. In addition, most of the study sites are in cities in southern China, leading to a lack of data from people in northern China, but subsequent studies will include a larger geographic range of people. What’s more, open label could lead to bias, and we expect that stratified analysis can balance some bias. Last but not least, the decision to select which investigational arm(s) to proceed to stage 2 is based on the analysis of safety and preliminary effectiveness at the end of 8 weeks of treatment in stage 1. However, it is a fact that early treatment outcome at the end of 8 weeks of treatment may not be related to relapse, and early safety at the end of 8 weeks of treatment does not fully represent long-term safety.

In conclusion, this adaptive trial has been launched recently and has been expected to search for new therapeutic strategies with stronger bactericidal activity, to contribute to the optimal dose of rifapentine in the Chinese population, and to inform on the feasibility of a treatment regimen using high-dose rifapentine with a shorter treatment duration for DS-TB.

## Supplementary Information

Additional file 1. Detailed schedule of evaluations/tests.

## Data Availability

Not applicable. This is a study protocol manuscript.

## Abbreviations

DS-TB: Drug-susceptible tuberculosis
TB: Tuberculosis
AEs: Adverse events
WHO: World health organization
FDA: Food and drug administration
NMPA: National medical product administration
PK/PD: Pharmacokinetic/pharmacodynamic
P: Rifapentine
H: Isoniazid
Z: Pyrazinamide
M: Moxifloxacin
SAEs: Serious adverse events
SRC: Safety reviewer committee
HIV: Human immunodeficiency virus
NMCID: National medical center for infectious diseases
CCO: Central coordinating office
CTCAE: Common terminology criteria for adverse events
mITT: Modified intention-to-treat
DST: Drug susceptibility testing
