# Supplementary material for: Optimizing (O) rifapentine-based (RI) regimen and shortening (EN) the treatment of drug-susceptible tuberculosis (T) (ORIENT) using an adaptive seamless design: study protocol of a multicenter randomized controlled trial
Source: BMC Infect Dis. 2023 May 8;23:300. doi: 10.1186/s12879-023-08264-2 (PMC10165810; doi:10.1186/s12879-023-08264-2)
Supplement: Supplementary file 1 — Additional file 1. Detailed schedule of evaluations/tests. [file 12879_2023_8264_MOESM1_ESM.docx]

**Additional file**: Detailed schedule of evaluations/tests

|  | Screening period | Baseline^1^ | Treatment period | | | | | | | | | | | | Post-treatment until 104 weeks after treatment initiation | | | | |
| --- | --- | --- | --- | --- | --- | --- | --- | --- | --- | --- | --- | --- | --- | --- | --- | --- | --- | --- | --- |
|  |  |  | intensive phase | | | | | | | | continuation phase^2^ | | | |  |  |  |  |  |
|  |  |  | 4d | 7d | 11d | 2w | 3w | 4w | 6w | 8w | 12w | 17w | 21w | 26w | 38w | 52w | 64w | 78w | 104w |
|  | V1 | V2 | V3 | V4 | V5 | V6 | V7 | V8 | V9 | V10 | V11 | V12 | V13 | V14 | V15 | V16 | V17 | V18 | V19 |
| Clinical assessment | | | | | | | | | | | | | | | | | | | |
| Demographic characteristics/medical history/height | X | ▲ |  |  |  |  |  |  |  |  |  |  |  |  |  |  |  |  |  |
| Signs and symptoms |  | X |  |  |  |  |  |  |  |  |  |  |  | X | X | X | X | X | X |
| Weight, concomitant medication | X | X | X | X | X | X | X | X | X | X | X | X | X | X |  |  |  |  |  |
| Eligibility evaluation | X | X |  |  |  |  |  |  |  |  |  |  |  |  |  |  |  |  |  |
| Central randomization |  | X |  |  |  |  |  |  |  |  |  |  |  |  |  |  |  |  |  |
| Adverse events | X | X | X | X | X | X | X | X | X | X | X | X | X | X |  |  |  |  |  |
| Bacteriological tests | | | | | | | | | | | | | | | | | | | |
| Respiratory specimen smear | X | ▲ |  |  |  | X |  | X | X | X | X | X | X | X | X | X | X | X | X |
| Respiratory specimen culture |  | X |  |  |  | X |  | X | X | X | X | X | X | X | X | X | X | X | X |
| Respiratory specimen Xpert^®^ MTB/RIF | X | ▲ |  |  |  |  |  |  |  |  |  |  |  |  |  |  |  |  |  |
| Respiratory specimen for rapid molecular test | X |  |  |  |  |  |  |  |  |  |  |  |  |  |  |  |  |  |  |
| Phenotypic susceptibility testing of Mtb bacterial isolate |  | X |  |  |  | X |  | X | X | X | X | X | X | X | X | X | X | X | X |
| Whole genome sequencing of Mtb bacterial isolate |  | X |  |  |  | X |  | X | X | X | X | X | X | X | X | X | X | X | X |
| Laboratory and auxiliary examinations | | | | | | | | | | | | | | | | | | | |
| Blood routine | X | ▲ | X | X | X | X | X | X | X | X | X | X | X | X |  |  |  |  |  |
| Liver function | X | ▲ | X | X | X | X | X | X | X | X | X | X | X | X |  |  |  |  |  |
| Renal function | X | ▲ |  |  |  | X |  | X | X | X | X | X | X | X |  |  |  |  |  |
| Electrolyte |  | X |  |  |  | X |  | X | X | X | X | X |  | X |  |  |  |  |  |
| Electrocardiogram | X | ▲ |  |  |  | X |  | X | X | X | X | X |  | X |  |  |  |  |  |
| Hemoglobin A1C |  | X |  |  |  |  |  |  |  |  | X |  |  | X |  |  |  |  |  |
| Chest CT | X | ▲ |  |  |  |  |  |  |  | X |  | X |  | X |  | X |  | X | X |
| Visual test | X | ▲ |  |  |  |  |  | X |  | X |  | X |  |  |  |  |  |  |  |
| Pregnancy testing, HBsAg, HIV and HCV antibody test | X |  |  |  |  |  |  |  |  |  |  |  |  |  |  |  |  |  |  |
| other | | | | | | | | | | | | | | | | | | | |
| PK/PD |  | within this interval | | | | | | | | |  |  |  |  |  |  |  |  |  |
| Blood sample storage^3^ |  | X |  |  |  |  |  |  |  | X |  | X |  | X |  |  |  |  |  |

1) For some collected data (▲) during the screening period, there is no need to repeat collection or conduct at baseline, and it can be used directly. Screening period and baseline laboratory examination can be collected before signing the informed consent, but it is required to be completed within 2 weeks before randomization.

2) For patients who completed treatment in 17 weeks, 26 weeks after randomization are taken as the first visit time after treatment completion.

3) The blood samples will be stored before treatment, at the end of intensive period and at the end of treatment.

4) The follow-up window of V2-V6 is ± 1 day, the follow-up window of V7-V11 is ± 3 days, and the follow-up window of V12-V19 is ± 1 week.
